# A cross-sectional study on breastfeeding practices and perceptions among mothers visiting a tertiary care hospital in India

**DOI:** 10.6026/973206300212267

**Published:** 2025-08-31

**Authors:** Sunil M. Doshi, Gunjita Gupta, Shreya Bose, Hirwa Daxini, Rucha Dave

**Affiliations:** 1 Department of Forensic Medicine, Dr N D Desai Faculty of Medical Science and Research, Dharmsinh Desai University, Nadiad, Gujarat, India; 2 Smt.B.K.Shah Medical Institute and Research Centre, Sumandeep Vidyapeeth University, Vadodara, Gujarat, India

**Keywords:** Breastfeeding, knowledge, attitude, practices

## Abstract

In India, cultural beliefs prevalent in numerous societies hamper the proper implementation of breastfeeding practices, which may adversely affect the normal development of infants. Therefore, it is of interest to assess breastfeeding awareness among mothers, emphasising on evaluating their knowledge, attitudes, and current breastfeeding practices as well as to compare findings with previous researches of a similar nature. Total 100 randomly selected mothers of infants visiting a tertiary care hospital were interviewed with predefined questionnaires. Questionnaires were based on assessment of knowledge, attitude and their breastfeeding practices. The study concluded that while the majority of mothers demonstrated adequate knowledge, positive attitudes, and appropriate breastfeeding practices, there are specific areas where maternal education still requires attention.

## Background:

Breast milk is a comprehensive source of essential nutrients such as carbohydrates, proteins, fats, vitamins, minerals, water, electrolytes, growth factors, enzymes, and hormones [1]. The World Health Organization (WHO) advocates for exclusive breastfeeding for the first six months of an infant's life, followed by continued breastfeeding alongside complementary foods up to two years of age or beyond [2]. WHO and United Nations Children's Fund (UNICEF) recommend initiating breastfeeding within an hour of life and the it should be given on demand that is as often as needed, day or night [3]. According to the American Academy of Paediatrics, breast milk is the preferred feeding for all infants, including full-term, premature as well as sick babies [4]. In India, cultural myths widespread across various societies hamper the proper implementation of breastfeeding practices, potentially affecting infant development adversely. One prevailing myth is related to colostrum, the yellowish, sticky breast milk produced towards the end of pregnancy, which is often believed to be harmful and causing gas and discomfort if fed to the newborn [5]. Contrary to this belief, WHO recommends colostrum as the ideal first food for newborns due to its rich nutritional content and immune-boosting properties [6]. Another misconception in Indian societies is that ill mothers cannot produce sufficient milk and should usually avoid breastfeeding during illness. However, WHO guidelines encourage breastfeeding even for HIV-infected mothers, alongside antiretroviral therapy, highlighting the importance of continued breastfeeding in various health conditions [7]. Different cultures in India have their own specific dietary opinions for lactating mothers. However, the Australian Breastfeeding Association proclaims that a balanced diet is generally sufficient to ensure an adequate milk supply, demystifying the belief that specific foods are required to boost milk production [8]. These cultural beliefs and misunderstandings accentuate the need for comprehensive education and awareness programs to promote optimal breastfeeding practices across diverse communities in India. The aim of this study was to evaluate mothers' awareness of breastfeeding with objectives to assess their knowledge, attitudes, and current practices. Therefore, it is of interest to report the findings of this study along with its comparison to those of previously published research articles of similar nature across the country to depict the diversity as well.

## Materials and Methods:

Present study was conducted at a tertiary care hospital of Gujarat, India. Approval from 'Local Institutional Ethics Committee' was obtained to conduct this study. Study population was consisted of the mothers who attended the pediatric outpatient department as well as immunization clinic. Selection of the mothers was randomized provided she had a one year old or younger child. Total duration of the study was six months. Consent for the participation in the study was taken from each individual. Data collection involved personal interviews with each mother using a structured questionnaire consisting of two pages. The first page gathered demographic information, while the second page contained 17 questions related to breastfeeding knowledge, attitudes, and practices, addressing both factual information and common misconceptions. The rationale of the study was explained to each participant, and questionnaires were translated into their vernacular language to ensure comprehension. A total of 100 samples was the study material. Design of the study was cross sectional with convenience sampling technique. Data were compiled using Microsoft Office Excel 2007 and presented in tabular format. Analysis was conducted using percentages to evaluate responses across different aspects of breastfeeding awareness and practices.

## Results:

Table 1 (see PDF) demonstrates demographic profile of the mothers included in the study. The present study highlights several key aspects of breastfeeding knowledge, attitudes, and practices among the surveyed mothers. Notably, while only a small percentage (4%) believed that exclusive breastfeeding should ideally last for 4 months, the overwhelming majority (96%) correctly identified that it should continue for at least 6 months from birth. All mothers (100%) recognized breastfeeding as crucial for optimal infant growth, immunity, and bonding between mother and child. There was a divided opinion regarding breastfeeding continuation during maternal illness, with 56% of mothers supporting cessation in such circumstances, while 44% disagreed. A significant proportion (91%) demonstrated awareness of proper latching techniques as well as concept of bonding (100%), crucial for effective breastfeeding. The majority (94%) were against offering plain water during exclusive breastfeeding, understanding its potential impact. Regarding formula feeding, a mere 12% believed it to be a better choice compared to breastfeeding, with 88% viewing breastfeeding as the easier and preferred method to nourish their child. Additionally, the vast majority (93%) was inclined to provide colostrum, recognizing its importance, and almost all (99%) were opposed to giving any form of pre-lacteal feed. Approximately 49% engaged in alternating the breasts during a single feeding session and 45% alternating the breasts across different feeding sessions with 6% doing both randomly. The majority (73%) practiced demand feeding with 27% followed scheduled sessions. An impressive 90% practised exclusive breastfeeding at the time of the interview. Initiation of breastfeeding was prompt for many mothers, with 48% initiating within the crucial first hour after birth, while 35% started within a few hours, and 17% commenced breastfeeding the day after birth. Health-wise, the vast majority (93%) reported no issues with breastfeeding, with only 2% experiencing sore nipples and 5% encountering insufficient milk supply, yet 97% did not discontinue breastfeeding due to these challenges. Regarding dietary practices to augment milk supply, approximately 48% did not introduce new food items, while 40% consumed "shira" (a solid mixture of flour, sugar, milk, and clarified butter), 7% consumed coconut, and 5% consumed "rab" (a liquid mixture of flour, jaggery, clarified butter, and water). The data from this study are compared with other similar studies, as shown in Table 2 (see PDF).

## Discussion:

Vijayalakshmi *et al.* included 122 mothers aged between 20 and 30 years, with the majority coming from rural backgrounds. Approximately half of the participants were either illiterate or had primary education [9]. Naseem Altaf *et al.* involved 400 mothers primarily aged 20-30 years, educated up to high school level [10]. Shwetal *et al.* focused on 175 postnatal mothers, most of who had secondary school education and were in the 20-30 age range [11]. Gadhavi *et al.* conducted research on 200 mothers, predominantly from rural areas, aged 15-25 years and educated up to high school [12]. Except few noticeable differences, the data from this study are comparable to these previous studies on different parameters. This study notes unexpected findings regarding mothers' perceptions of breastfeeding. It reveals a surprisingly high level of awareness among mothers regarding the benefits of breastfeeding. This awareness is attributed to their urban residence, as reported by many participants, or their visits to tertiary-level hospitals. Most mothers in the study displayed a positive attitude towards avoiding pre-lacteal feeding or formula feeding and endorsing colostrum. Additionally, they were observed to practice appropriate breastfeeding techniques.

While the majority of mothers possess adequate knowledge about the benefits of breastfeeding, there are still areas that require attention in their education. Specifically, there is a need to incorporate information about continuing breastfeeding during illness and to foster a positive attitude among mothers towards seeking advice from healthcare professionals when facing minor issues, to prevent premature cessation of breastfeeding. Concepts such as foremilk and hindmilk and the importance of fully emptying each breast during feeding sessions, should be taught during hospital visits. Health professionals should actively engage in promotional activities to ensure that mothers receive updated knowledge on various aspects of breastfeeding. According to the World Health Organization (WHO), preliminary feeds pose a danger because they substitute colostrum as the baby's first nourishment, thereby increasing the risk of infections such as diarrhoea, septicaemia, meningitis, and intolerance to proteins. A study conducted in Orissa, India, revealed that 100% of mothers practiced prelacteal feeding, with 55%, 50%, and 86% of mothers in industrial, urban, and rural communities, respectively, discarding colostrum [13]. Another study involving 1,050 mothers across 224 villages in central Karnataka found that prelacteal feeding was practiced universally (100%) [14]. The belief that colostrum should be discarded is a misconception found in many regions of India. A study involving 2,158 mothers from urban slums and rural areas of Maharashtra and Gujarat indicated that rural mothers tended to discard colostrum significantly less than urban mothers [15]. This highlights a cultural and regional difference in colostrum feeding practices. Another study concluded that discarding colostrum is not a widespread practice and varies based on cultural traditions and community norms [16].

Looking at global scenarios reveals some concerning trends in breastfeeding practices. Studies from Bangladesh and Kuwait indicate that only around 27% of mothers practice exclusive breastfeeding. Various factors contribute to this, including socio-demographic factors, workplace-related issues, and concerns about breast milk quantity [17, 18]. In Vietnam, data from a cross-sectional survey involving 6,068 mothers showed that 73.3% of newborns were given prelacteal feeds [19]. Similarly, a study in rural Egypt reported a 60% prevalence of prelacteal feeding [20]. Reasons cited for prelacteal feeding in Nigeria include delayed lactation and the apparent need to keep the baby's mouth moist and body warm [21]. In Bangladesh, a study found that 77% of infants received prelacteal feeds, influenced by socio-demographic factors and healthcare variables [22]. These findings highlight the complex challenges and varied practices surrounding early infant feeding across different regions and cultures globally. Regarding concerns about the nutritional value and quantity of breast milk, a study has suggested that human breast milk generally maintains a consistent composition and is minimally influenced by the mother's diet. The levels of carbohydrates, proteins, fats, calcium, and iron remain relatively stable, even if the mother's diet is deficient in these nutrients [23]. To increase breast milk production, many mothers use herbal galactogogues despite limited scientific evidence regarding their effectiveness and safety. Both herbal and pharmaceutical options have unreliable evidence supporting their ability to enhance breast milk production, with a lack of peer-reviewed studies proving their efficacy [24]. A systematic review concluded that due to insufficient evidence from some trials, no definitive recommendation can be made for the use of herbs as galactogogues. It emphasized the need for well-designed and well-conducted clinical trials that address the limitations of previous studies to establish robust evidence guiding recommendations on herbal galactogogues [25].

Evidence from a randomized controlled study highlights that counselling by trained professionals significantly increases the rate of sustaining exclusive breastfeeding, with over 80% of mothers counseled achieving this compared to 50% of those who were not [26]. A cross-sectional study focusing on rural populations suggests that tailored breastfeeding education during prenatal stages can enhance intentions and practices of exclusive breastfeeding, accentuating its positive impact on knowledge, attitude, and subjective norms while reducing perceived barriers [27]. In a study conducted in rural South India, a low rate of exclusive breastfeeding (48.5%) was attributed to perceptions of insufficient milk supply, prompting recommendations to evaluate the effectiveness of antenatal education in improving breastfeeding outcomes [28]. Similarly, research from Kashmir indicates a very low rate of exclusive breastfeeding (36%) as well, highlighting the need for increased educational and promotional efforts to enhance breastfeeding practices [29]. Overall, these studies emphasize the importance of comprehensive education and proactive promotional strategies to support breastfeeding practices among diverse populations.

## Conclusion:

While the majority of mothers demonstrate sufficient knowledge, positive attitudes, and appropriate breastfeeding practices, there are still specific educational areas that require attention. It is beneficial to implement institutional policies that regularly solicit feedback from mothers and assess their knowledge over time. This feedback can be utilized to adjust the content and methods of education programs. Such a proactive approach at the institutional level helps in continuously improving the quality and relevance of breastfeeding education, thereby supporting mothers in making informed decisions and achieving optimal breastfeeding outcomes.

## Source of funding:

Self

## Ethical clearance:

Approved by Institutional ethics committee
